# Collagen Scaffolds in Regenerative Endodontic Procedures: Current Evidence, Limitations, and Future Perspectives

**DOI:** 10.3390/polym18070894

**Published:** 2026-04-07

**Authors:** Qiong-Ling Shi, Xiao Zhu, Chen Chen, Jing-Yi Chen, Dan-Yang Lu, Ying Shi, Yan-Qi Chen, Zhi-Fang Wu

**Affiliations:** 1 Stomatology Hospital, School of Stomatology, Zhejiang University School of Medicine, Hangzhou 310006, China; shiql@zju.edu.cn (Q.-L.S.); 3210105264@zju.edu.cn (C.C.); 7322035@zju.edu.cn (D.-Y.L.); shiying2010@zju.edu.cn (Y.S.); 2 Zhejiang Province Clinical Research Center for Oral Disease, Key Laboratory of Oral Biomedical Research of Zhejiang Province, Cancer Center of Zhejiang University, Engineering Research Center of Oral Biomaterials and Devices of Zhejiang Province, Hangzhou 310006, China; 3 School of Stomatology, Zhejiang University, Hangzhou 310058, China; 22318748@zju.edu.cn; 4 Department of Anesthesiology, The First Clinical College, China Medical University, Shenyang 110001, China; padingboen@petalmail.com

**Keywords:** regenerative endodontic procedures, collagen scaffold, polymers, pulp-dentin complex, tissue engineering, clinical outcomes, histological characteristics

## Abstract

Predictable pulp-dentin regeneration continues to represent a major challenge in regenerative endodontic procedures (REPs). Although collagen-based scaffolds are widely investigated for their excellent biocompatibility, their ability to deliver consistent clinical and histological outcomes requires critical evaluation. This review summarizes recent advances in the application of collagen scaffolds for REPs. Clinical studies demonstrate that these scaffolds support high tooth survival rates and promote vitality recovery, root wall thickening, and apical closure. However, consistent root lengthening remains elusive. Histologically, the newly formed mineralized tissue from collagen scaffolds within REPs tends to be cementum-like or bone-like rather than reparative dentin, a pattern closely associated with the physicochemical properties of collagen, including pore size, porosity, concentration, stiffness, viscosity, and viscoelasticity. We conclude that while collagen scaffolds represent a “promising platform” for REPs due to their biocompatibility and clinical performance, current evidence indicates that they do not consistently achieve true pulp-dentin regeneration. We therefore propose targeted modification and advanced tissue engineering strategies to direct genuine regeneration. This review offers a framework for the rational design of next-generation collagen constructs toward more predictable regenerative outcomes.

## 1. Introduction

According to data from the World Health Organization databank covering the years 2000 to 2015, dental caries affects between 21% and 97.3% of children aged 5 to 12 years [1], often resulting in pulpal injury and necrosis [2]. The conventional management of immature permanent teeth with pulpal necrosis and apical periodontitis involves apexification and apical barrier techniques [3]. However, these methods do not succeed in restoring physiological pulpal function or facilitating root maturation.

Regenerative endodontic procedures (REPs), a biology-based therapeutic approach, aim to regenerate functional dental pulp tissue by inducing the differentiation of endogenous or exogenously introduced stem cells within the root canal, thereby promoting the continuous development of the pulp-dentin complex and tooth roots [4,5]. Typically, its clinical protocol mainly involves minimally invasive disinfection of the root canal system through chemical irrigation, followed by the induction of apical bleeding, and finally the achievement of hermetic coronal sealing using bioceramic materials [6]. Notably, when blood clot formation is insufficient, the rational combination of biomaterials can create optimized microenvironments that are conducive to regeneration, thereby supporting functional reconstruction through the use of growth factors and scaffold design [7]. These biomaterials can be broadly categorized into host-derived scaffolds (e.g., blood clots, platelet-rich plasma, platelet-rich fibrin), nature-derived scaffolds (e.g., collagen, hyaluronic acid, chitosan), synthetic scaffolds (e.g., synthetic hydrogels such as GelMA and peptide-based hydrogels), and bioceramic scaffolds (e.g., hydraulic calcium silicate-based materials including mineral trioxide aggregate and Biodentine) [8]. Nevertheless, selecting an appropriate scaffold remains a significant challenge, as essential cellular processes—such as migration, proliferation, and differentiation—are profoundly influenced by the properties of the materials used [9].

Collagen, as the primary component of dental pulp, exhibits viscoelastic properties akin to those of native pulp tissue [10], thereby garnering increasing interest in pulp tissue engineering applications [11,12,13]. In 2008, Jung et al. were among the pioneers in utilizing collagen matrices in REPs to promote new tissue formation within the pulp chamber, particularly when inadequate bleeding hinders natural clot formation [14]. Subsequently, a growing number of studies have employed collagen as a carrier for stem cells and growth factors, highlighting its favorable biocompatibility and potential to facilitate tissue regeneration in dental pulp [15,16,17,18]. A significant advantage of collagen in REPs is its established application in human medicine, with several commercially available products—such as CollaPlug^®^, CollaTape^®^, Bio-Gide^®^, SynOss^TM^ Putty and CollaCote^TM^—produced on a large scale [17,18,19,20,21]. Studies have reported the formation of pulp-like connective tissues from collagen scaffolds in REPs within experimental models [15,22,23]. However, the structural and functional characteristics of these tissues warrant further investigation. Furthermore, it must be noted that most commercially available collagen products currently employed in REPs originate from non-pulp environments, raising legitimate concerns about their appropriateness for pulp regeneration.

Given the role of collagen scaffolds in REPs, this narrative review summarizes recent advances in their applications for REPs, with a focus on clinical outcomes and histological evaluations. Considering the current limitations of collagen scaffolds in REPs, we further discuss key physicochemical properties and innovative tissue engineering strategies to optimize collagen scaffolds. This work focuses on synthesizing current evidence, critically analyzing limitations, and proposing targeted future directions based on the literature, aiming to guide the rational design and clinical translation of next-generation collagen scaffolds in REPs.

## 2. Review Methodology

Databases searched: PubMed, Scopus, and Web of Science.

Search terms: combinations of “revascularization”, “revitalization”, “regenerative endodontics”, “pulp revascularization”, “pulp regeneration”, “dentin regeneration”, “regenerative endodontic therapy”, “regenerative endodontic procedures”, “regenerative endodontic treatment”, “REPs”, “collagen”, “collagen scaffold”, “collagen membrane”.

Inclusion criteria: peer-reviewed English articles published up to October 2025, including clinical trials, observational studies, case series, animal studies, and in vitro experiments focused on collagen scaffolds in REPs.

Exclusion criteria: studies not related to collagen-based scaffolds for pulp/dentin regeneration, conference abstracts, letters, and opinion articles.

Analytical framework: owing to the high heterogeneity among included studies (varying study designs, scaffold types, outcome measures, and follow-up periods), we performed a thematic evidence synthesis organized by clinical outcomes, histological observations, physicochemical properties, and tissue engineering strategies. We also emphasize that clinical, animal, and in vitro evidence is discussed within its respective hierarchical context to maintain interpretive validity and scientific rigor. And clinical studies (especially randomized controlled trials) are prioritized for guiding clinical recommendations, while animal and in vitro studies provide exploratory insights for future research.

## 3. Clinical Application Outcomes of Collagen Scaffolds in REPs

**Figure 1:**
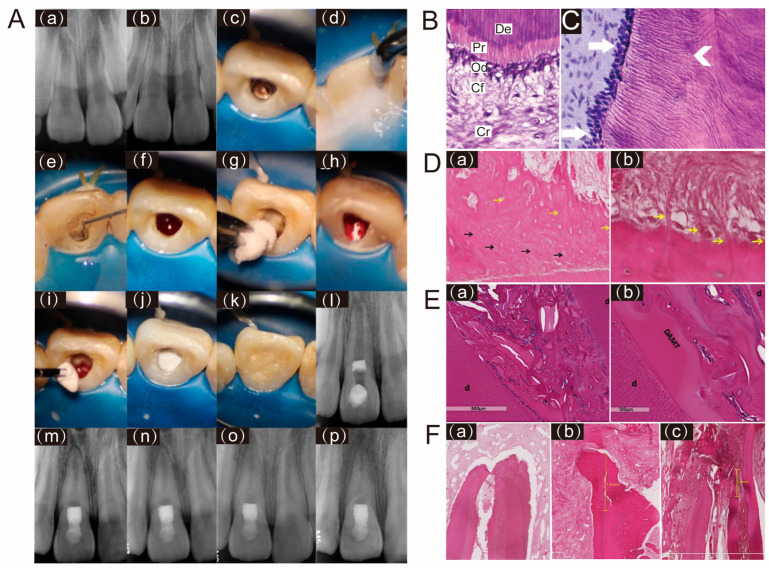
Clinical diagram and histological evaluation of regenerative endodontic procedures (REPs) using collagen scaffold. (**A**) REPs performed with a pure collagen scaffold for an immature tooth 8 with intrusive luxation and diagnosed as symptomatic apical periodontitis approximately 4 months after injury. The tooth showed continued root canal space narrowing 48 months after treatment. (**a**,**b**) Periapical radiographs of tooth 8 after injury and 4 months post-injury. (**c**–**e**) Clinical image of tooth 8 involved access opening, irrigation, and medication of the root canal. (**f**) Clinical image of inducing apical bleeding into the canal space. (**g**,**h**) Clinical image of placing a piece of resorbable collagen sponge over the blood clot. (**i**,**j**) Clinical image of incrementally placing a 3 mm thick layer of bioceramic paste over the collagen sponge. (**k**) Clinical image of filling the access cavity with resin composite. (**l**–**p**) Periapical radiographs of tooth 8 after treatment, 6-month, 12-month, 24-month, and 48-month follow-up. Adapted from [11], Elsevier, 2022. (**B**) HE staining of the dentin-pulp interface in a healthy tooth. Dentin (De) with tubules running parallel to each other; predentin (Pr) with uniform thickness; palisading odontoblast layer (Od); cell-free zone (Cf); cell-rich zone (Cr) (original magnification ×400). Adapted with permission from [69], Elsevier, 2017. (**C**) Reparative dentin represents true dentin regeneration. Mature, secretory odontoblasts lining newly formed tubular dentin (arrows). Adapted with permission from [50], Elsevier, 2018. (**D**) Cementum-like tissue. (**a**) deep layer of acellular cementum-like tissue (black arrows) covered by cellular cementum-like tissue (yellow arrows). (**b**) Sharpey’s fibers-like projections are inserted into a cementum-like layer (yellow arrows). Adapted with permission from [62], Elsevier, 2017. (**E**) bone-like tissue. (**a**) The newly formed intracanal mineralized tissue that is transitioning toward full calcification is intermixed with scaffold particles and connective tissue. (**b**) The areas of solidification between dentinal walls, dentin-associated mineralized tissue (DAMT), and the newly formed intracanal mineralized tissue with calciotraumatic lines in between. Adapted with permission from [52], Elsevier, 2019. Note: While cementum-like tissue (**D**) and bone-like tissue (**E**) can contribute to root wall thickening and apical closure (positive clinical surrogates), these structures constitute a tissue repair response, rather than true functional regeneration of the native pulp-dentin complex. (**F**) periapical hard tissue. (**a**,**b**) The apical foramen was sealed by cementum-like tissue; (**c**) the apical foramen was closed by bone-like tissue. Adapted with permission from [62], Elsevier, 2017.

Since the introduction of collagen scaffolds within REPs in 2008 [14], clinical applications have largely relied on commercial products, as detailed in Table 1. Numerous clinical studies and systematic reviews have validated the effectiveness of collagen scaffold in REPs, with survival rates ranging from 85% to 100% and success (healed) rates from 80% to 100% over a follow-up period of 23 months [16,18,24,25] (Table 2). Similarly, for necrotic immature teeth, REPs employing BCs or other scaffolds demonstrated survival rates ranging from 95.6% to 100% at least a 12-month follow-up [25,26,27,28]. These outcomes indicate that collagen scaffolds yield clinical success rates comparable to those of other scaffolds in REPs. Consequently, both the American Association of Endodontists (AAE) and Chinese expert consensus guidelines recommend their use in REPs [4,6], particularly in cases where adequate blood clot formation is compromised (Figure 1A). Nevertheless, the long-term effectiveness of collagen scaffolds in REPs warrants further validation through more rigorous clinical studies.

The success of REPs is not solely defined by the resolution of clinical signs and the evidence of radiographic healing; it also encompasses the potential to promote continued root development and apical closure. A previous study has highlighted the significant role of collagen scaffolds in promoting the thickening of dentinal walls during REPs [29]. Similarly, Jiang et al. also found that the Bio-Gide^®^ collagen membrane significantly increased dentin wall thickness in the middle third of the root after REPs compared to BCs [30]. An increase in root thickness from 3.0 mm to 5.0 mm enhances fracture resistance by 70% [31]. Thus, these findings underscore the importance of root thickness in improving the mechanical properties of dental structures.

One randomized controlled clinical trial reported that REPs utilizing collagen scaffolds were found to induce apical foramen closure in 47% of teeth; this process began as early as 6 months postoperatively, with closure achieved in the vast majority (96%) of cases by 24 months [25]. Interestingly, another study suggested that platelet-rich fibrin (PRF) might possess superior regenerative capacity compared to collagen scaffolds, as evidenced by increased apical closure, dentin thickness, and root lengthening [32], potentially due to its higher concentration of growth factors. However, a meta-analysis concluded that no significant difference exists among BCs and other exogenous scaffolds in terms of root development outcomes [33], whereas mounting clinical evidence indicates that 67.3% to 82% of teeth presented with root canal wall thickening [16,18,25,29,34,35] (Table 2). Specifically, randomized controlled trials have quantitatively confirmed that collagen membranes (e.g., Bio-Gide^®^) significantly increase dentin wall thickness in the middle third of the root after REPs, compared with the blood clot scaffold (*p* < 0.05) [25,30]. It implies that the advantage of collagen scaffolds could be specific to enhancing dentin wall thickness [17,25]. Key prognostic factors such as the tooth’s developmental stage, follow-up duration, and disease etiology significantly influence the outcome of root development [18,36]. According to the clinical considerations for REPs revised by AAE in 2023 [4], an increase in the thickness of the root canal walls is typically observed 12 to 24 months following treatment. However, it is worth noting that the study, with a maximum follow-up of only 12 months, might be insufficient to fully capture this outcome [32]. Consequently, the long-term stability and potential changes in outcomes over an extended period remain an open question.

Regarding the pulp vitality recovery after REPs, the rate of positive responses to the cold test and/or electric pulp testing (EPT) on necrotic immature permanent teeth ranges from 14.5% to 33.3% [28,37]. For REPs utilizing collagen scaffolds in such teeth, the reported positive response rate ranges from 32% to 60%, with the majority occurring within 24 months [16,18,25]. These findings suggest that the use of collagen scaffolds within REPs may be associated with a higher rate of pulp vitality recovery, which aligns with the results reported by Jiang et al. [25]. In contrast, several studies using collagen matrices in REPs for immature permanent teeth reported no pulp sensibility responses throughout the follow-up period [38,39,40]. Several factors may explain these negative outcomes [41]: (1) the inherent difficulty in obtaining reliable pulp testing scores in pediatric patients; (2) potential interference from a thick, multilayered coronal seal placed over the scaffold; and (3) the absence of well-organized dentin tubules in newly regenerated and mineralized root canal tissues. Unlike natural dentin, where sensitivity is mediated by hydrodynamic activity associated with A-β sensory fibers [42], the regenerated tissue lacks this structural basis for normal sensitivity. Thus, the aforementioned hypotheses may collectively account for the generally lower rate of positive sensitivity responses observed in REPs, underscoring the need for cautious interpretation of pulp sensibility test results in this context.

Root canal calcification is a frequently reported complication following REPs, and its incidence ranges from 30.7% to 62.1% with an average follow-up period of 12 to 24 months [17,35,43,44,45]. For REPs utilizing collagen scaffolds, Lin et al. reported that the calcification rate was 37.6%, with most cases detected at the 6-month recall [46]. Despite extended follow-up durations ranging from 15 to 33 months, the calcification rate remained stable at approximately 47% in REPs with collagen scaffolds [25], indicating that not all treated teeth develop calcification, even with follow-ups as long as 78 months [17]. However, Jiang et al. found no significant correlation between root canal calcification and factors such as tooth type, etiology, preoperative diagnosis, apical lesion status, initial root development stage, intracanal bleeding quality, or scaffold type [17]. It is noteworthy that induced apical bleeding—a procedural step common to most REPs prior to the placement of PRF, collagen, or other scaffolds—may introduce periodontal and bone marrow-derived stem cells into the root canal space [47]. Biological evidence suggests that these cells can promote the formation of bone- or cementum-like mineralized tissues in the canal space [47]. Therefore, while scaffold type was not identified as a correlative factor, the potential contribution of the initial BCs to calcification cannot be entirely excluded and merits further validation in prospective studies.

In summary, despite the fact that most commercially available collagen products are mainly applied in non-pulp environments (Table 1), their use in REPs has shown potential, as evidenced by high survival/success rates and pulp vitality recovery, as well as promoting dentinal wall thickening and apical closure in necrotic immature teeth (Table 2). However, most studies were retrospective in design and thus had certain limitations. Assessments of root development based on two-dimensional radiographs are inherently less accurate and comprehensive compared with quantitative methods of CBCT. Thus, the marked heterogeneity across different studies made it difficult to accurately assess the actual role and efficacy of collagen scaffold in REPs. Moreover, some researchers are concerned that limitations, such as rapid degradation and low mechanical strength, may affect the performance of collagen-based scaffolds in REPs [48]. Several histological studies of REPs with collagen scaffold on human teeth have reported no detectable collagen residues as early as 5.5 months post-treatment [49,50,51]. In cases using SynOss™ Putty—a composite containing both collagen and hydroxyapatite—histologic sections revealed residual scaffold particles at 7.5 months after REPs, though without evidence of remaining collagenous material [52]. While no available studies have reported that collagen degrades too rapidly to support tissue regeneration in REPs, the degradation rate of current commercial collagen-based scaffolds has not been systematically evaluated in long-term clinical studies. Thus, the adequacy of their degradation rate remains to be confirmed by further research focusing on the synchronization between scaffold degradation and new tissue formation. Similarly, none of the clinical studies or cases reported failures attributable to poor mechanical properties of collagen scaffolds, which suggests that the mechanical performance of existing commercial collagen scaffolds may be sufficient for the short- to medium-term requirements of REPs [53]. However, this does not constitute definitive evidence of adequacy, as long-term mechanical stability and performance under physiological conditions have not been fully evaluated. Theekaku et al. reported a failure rate of 10% (13/120 teeth) in REPs using collagen, with primary causes including persistent infection, coronal and/or root fracture, and postoperative secondary trauma [18]. Thus, future efforts should focus on developing multi-functionally enhanced collagen composites that improve anti-bacterial abilities, coupled with prospective long-term clinical studies to validate sensory recovery and minimize mineralization risks [54].

**Table 1:** Commercially available collagen products applied in REPs.

Brand Name	Character	Main Composition	Source	Main Uses in Dentistry	Clinical Outcomes in REPs
CollaPlug^®^	Collagen sponge	High-purity Type I collagen	Bovine	Hemostasis, oral wound protection and repair, and small bone defect support	Radiographic periapical healing, root development in root length and dentin wall thickness, pulp vitality recovery [24]
CollaCote™	Collagen sponge	High-purity Type I collagen	Bovine	Hemostasis, oral wound protection, and repair	Radiographic periapical healing, root development, especially in increased root length and dental wall thickness, and apical closure [27]
CollaTape^®^	Collagen sponge	High-purity Type I collagen	Bovine	Guided tissue regeneration (GTR), oral wound repair, and protection	Radiographic periapical healing, root development, pulp vitality recovery [19]
Bio-Gide^®^	Bilayer membrane	Cross-linked Type I/III collagen	Porcine	GTR, guided bone regeneration (GBR)	Radiographic periapical healing, root development, especially in increased wall thickness, pulp vitality recovery [25]
SynOss™ Putty	Bone graft substitute	Type I collagen and hydroxyapatite composite	Bovine	Regenerative endodontic procedures (REPs), GBR, and alveolar ridge preservation	Radiographic periapical healing, partial or complete root mineralization [52]

**Table 2:** Characteristics of clinical studies using collagen scaffolds in REPs.

Authors	Research Type	Scaffold Type	Diagnosis	Age (Years)	Tooth Maturity	Sample Size	Irrigant and Medicament Used	Follow-Up Duration (Mons)	Clinical Outcomes		
									Primary Outcome (Clinical Success Rate)	Secondary Outcomes	Tertiary Outcome
Jiang 2017 [30]	RCT	Bio-Gide vs. Blood clots	AP (14%) CP (86%)	9.82 ± 1.5 10.3 ± 1.9	Immature	43	NaOCl 1.25% +EDTA 17% +Ca (OH)_2_	16.1 ± 8.8 15.0 ± 5.8	100%	Root length ↑ Root width (apical and middle) ↑ AD ↓	EPT 33% vs. 18%
Rizk 2019 [27]	RCT	Collacote vs. PRP	PN, with or without periapical lesions	9.08 ± 1.04	Immature	26	NaOCl 2% +EDTA 17% +TAP	12	100%	Root length ↑ Root width ↑ Periapical density ↑ AD ↓	No response
Chrepa 2020 [24]	Retrospective study	CollaPlug	PN, with or without periapical lesions	7–26	Immature	51	NaOCl 1.5% (55%) 6% (45%) +EDTA 17% +TAP (25%) /DAP (33%) /Ca (OH)_2_ (42%)	25.2	84.3%	Root development	EPT and/or CPT 54%
Mittal 2021 [34]	RCT	Collagen vs. Collagen + PRP	PN, with or without periapical lesions	12.66 ± 4.47	Immature	31	NaOCl 5.25%; +EDTA 17% +TAP	12	88% Vs 85.7% *p* > 0.05	Root length ↑ Root width ↑ AD ↓	No response
Zeng 2022 [55]	Retrospective study	collagen	AP (81.9%) AA (18.1%)	10.3 ± 0.7	NA	116	NaOCl 1.5% +EDTA 17% +TAP	12–60	99.1%	Root length ↑ (93.1%) Root width AD (81%) ↓	NA
Jiang 2022 [25]	RCT	Bio-Gide vs. Blood clots	PN (7%) AP (93%)	10.6 ± 1.7 11.0 ± 1.9	Immature	76	NaOCl 1.25% +EDTA 17% Ca (OH)_2_	33.1 ± 21.8 28.1 ± 18.6	100%	Root length ↑ Root width (middle 1/3) ↑ Root width (apical 1/3) ↑ AD ↓	EPT 32% vs. 18%
Lu 2023 [21]	Retrospective study	CollaCote	PN	28.5 ± 12.4	Mature	37	NaOCl 1.5% +EDTA 17% +Ca (OH)_2_	52.0 ± 23.35	89.2%	NA	EPT and/or CPT 35.1%
Jena 2023 [29]	Prospective clinical observational study	collagen	PN, with or without periapical lesions (NA)	9	Immature	30	NaOCl 5.25% +EDTA 17% +TAP	24	93%	Root length ↑ (93%) Root width ↑ (82%) AD (65.8%) ↓	No response
Theekakul 2024 [18]	Retrospective study	CollaPlug	PN, with or without periapical lesions	<12 (63.3%) >12 (36.7%)	Immature	120	NaOCl 1.25–5.25%; +EDTA 17% +Ca (OH)_2_/TAP	12–148	80%	Root length ↑ Root width ↑ AD ↓	EPT and/or CPT 41.7%
Dave 2025 [16]	Retrospective study	CollaPlug vs. ACM + CollaPlug, /CollaTape/CollaCote	DA (10%) AP (70%) AA (30%)	14.1 ± 7.5 15.3 ± 7.5	Immature	41	NaOCl 1.5–6.0% +EDTA 17% +Ca (OH)_2_	6–86	80% vs. 85.7%	Root development 90% vs. 90.5%	EPT and/or CPT 60% vs. 42.9%
Sultan 2025 [32]	RCT	Collagen/PRF/HA	PN	10–18	Immature	45	NaOCl 1.5% +TAP	12	100%	Root length ↑ Root width↑ AD ↓	NA
Song 2025 [35]	Retrospective study	collagen	AP (60%) AA (40%)	≤8 (12.9%) ≤15 (87.1%)	Immature	101	NaOCl 2.5% +EDTA17% +Ca (OH)_2_	42	85.15%	Root length ↑ Root width ↑ AD (67.33%) ↓	NA

Abbreviations: RCT: randomized controlled trial; PN: pulp necrosis; AP: apical periodontitis; CP: chronic pulpitis; AA: apical abscess; AD: apical diameter; Ca (OH)_2_: calcium hydroxide; EPT: electric pulp test; CPT: cold pulp test; DAP: double antibiotic paste; TAP: triple antibiotic paste; ACM: amnion–chorion membranes; ↑: arrow indicates upregulation; ↓: arrow indicates downregulation.

## 4. Characteristics of the Formed Mineralized Tissue in REPs with Collagen Scaffolds

The properties and quantity of mineralized tissue within the root canal are closely related to the prognosis of REPs. Although clinical and imaging studies have demonstrated promising outcomes of REPs utilizing collagen scaffolds, the structural and functional characteristics of the newly formed mineralized tissue in the root canal warrant further investigations.

Based on the formation mechanisms, the newly formed mineralized tissues within the root canal after REPs can be primarily categorized into four distinct types: reparative dentin, cementum-like tissue, bone-like tissue, and periapical hard tissue [56] (Figure 1C–F). To date, cementum-like tissue and bone-like tissue remain the dominant mineralized tissues detected in the root canal after REPs [56,57], indicating that most scaffold materials applied in current REPs trigger a tissue repair process instead of genuine pulp-dentin complex regeneration. Repair is defined as the formation of cementum-like and bone-like tissue that restores structural integrity but fails to recover full biological function, while true regeneration entails the de novo formation of a fully functional pulp-dentin complex, characterized by reparative dentin deposition, reinnervation, and functional vascularization.

Cementum-like tissue, also known as dentin-associated mineralized tissue (DAMT), is formed through the differentiation of stem cells within the periapical tissues, following the apical blood supply [58,59]. DAMT manifests as mineralized tissue with a relatively uniform thickness, which may or may not contain embedded cells within the mineralized matrix (Figure 1D). The boundary between cementum-like tissue and canal dentin can be clearly identified by the absence of dentinal tubules in the former [57]. The bond between DAMT and the dentin wall is not particularly robust; certain regions are detached from the wall, while others are anchored to the dentin wall via Sharpey’s fiber-like tissue [23,49,57] (Figure 1D(b)). Longitudinal sections of teeth in humans treated with CollaPlug^®^ after REPs demonstrated that the newly formed mineralized tissue on the canal walls comprised both cellular and acellular cementum-like tissue, as well as bone-like tissue [49]. Furthermore, the canal dentin appeared to connect directly to the cementum-like tissue, with collagen bundles inserted into both the cementum-like and bone-like tissue at right angles, resembling Sharpey’s fibers [49]. Some researchers have proposed that specific demineralization treatments of dentin, such as increased ethylenediaminetetraacetic acid (EDTA) irrigation, could create a hair-like protrusion structure on the dentin surface, thereby enhancing the attachment strength of newly mineralized tissue to the root walls [56,57]. Additionally, other researchers have indicated that DAMT continues to deposit over time until the root canal is completely sealed [60]. However, the relatively short follow-up periods in both animal and clinical studies conducted thus far provide insufficient evidence to conclusively validate this assertion. Elnawam et al. have found that in the REPs for necrotic mature canine teeth, cementum-like tissue deposition was detected on internal canal walls of most samples in the BCs group and bovine dental pulp-derived extracellular matrix (P-ECM) hydrogels group [22]. However, intracanal hard tissue detected was significantly higher in the BCs groups compared to the P-ECM group [22]. Similarly, another study also established a model of REPs for necrotic mature canine teeth and found that, there was no regenerated mineralized tissue on the root canal dentin in most roots in the BCs and CollaPlug^®^ groups and all roots of the amnion-chorion membrane (ACM) group, although the amount of regenerated fibrous tissue and the perfused blood vessels in the root canals was greater in the membrane groups than in the BCs group [23]. This might be due to the retained growth factors and ECM components within the collagen scaffold that could influence the chemotaxis and commitment of surrounding stem cells [61].

Unlike DAMT, bone-like tissue, also referred to as bony islands (BI), is situated within the inner lumen, independent of the dentin wall [56]. These tissues manifest as islands of mineralized matrix that harbor numerous embedded cells, blood vessels, and bone marrow-like tissues [57] (Figure 1E). A histological study revealed that bone-like tissue comprised osteocyte-like and osteoblast-like cells, which contributed to the formation of mineralized tissue islands within the central portion of the canal space [49]. In certain specimens, it was observed that BI potentially originated from the bone marrow in the periapical area [62] (Figure 1F(c)) and occasionally appeared to connect with DAMT [52] (Figure 1E(a)). Additionally, several areas of fusion and calciotraumatic lines could be observed between BI and DAMT [52] (Figure 1E(b)). Another case in a canine animal model exhibited periodontal ligament (PDL)-like tissue, albeit very loose, located between the BI and the DAMT, resembling the naturally occurring structure on the outer root wall [22]. It is conceivable that PDL cells migrating along the dentin walls and bone marrow cells compete for space within the root canal lumen, with different cell types potentially being directed towards their preferred microenvironments to form their respective tissues.

According to the three-level outcome criteria for REPs established by the AAE, some researchers have proposed that, given the current immaturity of REP techniques, the presence of cementum-like tissue within the root canal could already satisfy the first- and second-level criteria and achieve a favorable prognosis [46]. The literature indicates that the prognosis of cementum-like tissue is superior to that of bone-like tissue [63]. However, the most desirable outcome remains the formation of reparative dentin [56]. Studies indicate that collagen scaffolds could promote the formation of reparative dentin (Figure 1C). Abdelsalam et al. found that, compared to the BCs group, the collagen in the BCs group exhibited clearly defined, distinct cellular elements and new dentin formation at the pulpal side of the root dentin in a canine animal model [15]. Furthermore, decellularized extracellular matrix from the periapical lesion (PL-dECM), along with periapical lesion-derived stem cells (PLDSCs), was implanted into the subcutaneous area of nude mice, resulting in an ideal pulp-like matrix characterized by a relatively dense eosin-stained matrix with numerous ordered nuclei and a predentin-like structure adjacent to the tooth section [64]. Immunohistochemical staining further demonstrated that PLDSCs within PL-dECM exhibited higher protein expression levels of dentin Sialophosphoprotein (DSPP), dentin matrix protein 1 (DMP-1), and vascular endothelial growth factor (VEGF) [64]. Another study employing both hematoxylin-eosin staining and immunostaining revealed that the neotissues formed represented dentin-like tissue in teeth treated with CollaPlug^®^ in REPs [50]. The newly formed dentin appeared organized and tubular, representing a primary dentin phenotype. Moreover, the newly formed dentin was continuous with native dentin and was lined with mature, secretory odontoblast-like cells [50]. This finding is unique and novel, as previous studies investigating the nature of neotissues observed a reparative dentin phenotype that differs from native dentin in terms of volume and density [65,66].

In addition, a study investigated the efficacy of collagen scaffolds with varying degrees of blood draw in humans, suggesting that collagen scaffolds alone are insufficient to support pulp-like tissue formation [52]. In teeth treated with SynOss^TM^ Putty and blood, histological examination revealed the formation of intracanal mineralized tissue around the scaffold particles, which solidified with newly formed cementum-like tissue on the dentinal walls. In contrast, teeth treated with SynOss^TM^ Putty and minimal bleeding (limited to the apical third) exhibited newly formed tissues only in the apical area, and the remaining root canal spaces were filled with disintegrating SynOss^TM^ Putty particles. These results suggest that the observed regenerative/reparative outcomes in REPs are likely due to a synergistic interaction between the collagen scaffold and the cellular and molecular components introduced by induced apical bleeding. The blood supplies a rich source of stem cells (e.g., from the apical papilla and bone marrow) and growth factors, and the collagen scaffold acts as a biomimetic matrix to support stem cell adhesion, proliferation, and differentiation, and to sustain the release of growth factors [67].

For the changes to the periapical and periodontal tissues, histological studies have indicated that the apical development appeared to be an extension or growth of the cementum/DAMT structure [15,52,57] (Figure 1F), facilitating apex closure or the reconstruction of normal apex anatomy. This newly formed tissue is continuous with the PDL at the apex, where the scaffold material is absent [15,49,52]. Additionally, PDL-like fibers are inserted into the cementum-like and bone-like tissue at right angles as Sharpey’s fibers [49]. Evidence from a study demonstrated the presence of cementocytes within the apical apertures and apical closure with cementum-like tissue in the BCs and collagen group. In contrast, the BCs group exhibited sporadic degenerative alterations alongside heavily pigmented basophilic condensation of fibrous tissues [15]. The inability to achieve complete sealing of the apex is frequently associated with BI [57,68] (Figure 1F(c)), and the presence of BI may contribute to the higher failure rate of REPs compared to apexification in immature permanent teeth [46].

In summary, although clinical and imaging investigations have documented positive outcomes for REPs utilizing collagen scaffolds, histological analyses have consistently indicated a tissue repair process, marked by the formation of cementum-like or bone-like tissue, rather than true regeneration of a functional pulp-dentin complex with structured reparative dentin and functional innervation. Indeed, cementum-like tissue and bone-like tissue remain the predominant mineralized tissues identified in the root canal following REPs using most conventional scaffold materials, including BCs and PRF [56,57]. Among these alternatives, collagen scaffolds and PRF typically trigger more mineralized tissue deposition with well-organized, highly vascular connective tissue relative to BCs [32,52]. However, direct comparative histological evidence in human specimens is scarce, and most of the above comparative findings are derived from preclinical in vitro and in vivo models. Accordingly, the current limitation of collagen scaffolds to reliably drive targeted dentinogenesis in REPs highlights a critical scientific and translational gap that must be prioritized and addressed in future biomaterial design and clinical research.

## 5. Physicochemical Properties of Collagen Modulating the Formation of the Pulp-Dentin Complex

The proliferation and differentiation of stem cells (such as dental pulp stem cells, DPSCs) within a specific microenvironment serve as an indispensable link in the formation of the pulpal-dentin complex [70,71]. Understanding the relevant physicochemical properties of biomaterials is crucial for optimizing the survival microenvironment of stem cells, thereby maximizing the efficacy of cell-based therapies. Collagen scaffolds, primarily composed of type I collagen, are regarded as effective substitutes for the extracellular matrix (ECM) due to their porous physicochemical structure and preserved sequences capable of binding to specific cell recognition sites, such as the RGD (Arg-Gly-Asp) sequence [71,72]. During dental pulp regeneration, integrins on stem cells mediate cell-collagen adhesion to form focal adhesions, which subsequently connect with the intracellular cytoskeletal protein vinculin to establish a mechanism capable of transmitting mechanochemical signals across the cell membrane through conformational changes. This establishes a dynamic linkage capable of sensing microenvironmental rigidity variations and modulating the expression of stem cell markers [73]. Meanwhile, the porous physicochemical structure of collagen scaffolds provides essential spatial accommodation for stem cell adhesion and proliferation [72,73]. Collagen scaffolds are considered promising carriers for stem cells and growth factors in dental pulp regeneration, demonstrating favorable biocompatibility and regenerative potential that hold significant promise for future applications [74,75,76,77]. Nevertheless, the influence of their intrinsic physicochemical properties on regenerative outcomes remains insufficiently elucidated, necessitating further investigation to inform standardized design and clinical translation.

Pore size is often the primary consideration among various physicochemical parameters due to its direct influence on cell infiltration, nutrient diffusion, and vascularization. The influence of pore size on cellular behavior is size-dependent and presents a biological trade-off [78]. Small pores (20–50 μm) increase the surface area available for initial cell attachment and aggregation but restrict cell migration. Scaffolds dominated by mi-croporosity often limit cellular infiltration to the periphery, leading to premature occlusion. This physical barrier impedes the diffusion of oxygen and nutrients to the interior and the removal of metabolic waste, often resulting in a necrotic core and failure of deep tissue regeneration. In contrast, macropores (>100–300 μm) support rapid cell migration and are essential for endothelial cell infiltration and capillary network formation. Adequate pore size facilitates angiogenesis by enabling the transport of signaling molecules such as VEGF and FGF2, which are critical for guiding vascularization in engineered pulp tissue [78,79]. Multiple investigative groups have reported superior regenerative outcomes where pore size was maintained within an optimal window of 60–90 μm, particularly for mesenchymal stem cells (MSCs) with an average diameter of 30 μm [80]. For instance, Qianli Zhang et al. prepared collagen scaffolds with varying average pore sizes (approximately 20 μm, 65 μm, and 145 μm) and demonstrated that the group of 65 μm induced the highest levels of odontogenic-related gene (DSPP, DMP-1) and protein (DMP-1) expression in human dental pulp cells (hDPCs), simultaneously promoting mineralization and vascularized tissue formation [81]. Similarly, Hengameh Bakhtiar et al. reported that ECM scaffolds with a pore size of 78.04 ± 16 µm exhibited superior physicochemical properties, with higher mineralization nodule formation and calcium deposition compared to other pore size groups, and more significantly supported the migration behavior of human dental pulp stem cells (hDPSCs) [82]. Furthermore, the porosity of collagen scaffolds is critical for achieving pulp regeneration, highlighting its significance in scaffold-based pulp regeneration strategies. Generally, higher porosity facilitates vascularization and nutrient transport. However, excessively high porosity often significantly reduces the mechanical strength of collagen scaffolds, potentially failing to maintain the stability of the pulp cavity structure and thus hindering pulp regeneration. Currently, collagen scaffolds used in pulp therapy typically have a porosity controlled at around 95.6% [83].

The pivotal role of collagen scaffold concentration in steering the regenerative process toward functional dentin-pulp complex formation has been well-documented. As one study demonstrated, while low-density collagen matrices are susceptible to severe contraction, appropriately increasing the concentration reduces shrinkage and enhances both cellular distribution and odontogenic differentiation within simulated root canals. However, surpassing the optimal density creates physical barriers that severely restrict cell migration, proliferation, and overall survival [84]. Another study by Hengameh Bakhtiar et al. reported that decellularized human amniotic membrane (HAM) scaffolds at 3.00 mg/mL displayed enhanced degradation and significantly promoted the migration of hDPSCs compared to lower-concentration variants [82]. Subsequent animal studies demonstrated that lyophilized ECM scaffolds at a concentration of 3.00 mg/mL exhibited optimal performance, showing the highest viability and proliferation rates of human bone marrow mesenchymal stem cells (hBMMSCs) along with marked upregulation of dentinogenic markers such as DMP-1 and collagen I [85]. However, no consensus has been reached regarding a well-defined optimal concentration range, underscoring the need for further systematic investigation. This discrepancy could be due to differences in the evaluation criteria established for the experiments, or alternatively, to the influence of other physicochemical properties of the collagen scaffold.

As a key microenvironmental cue, the stiffness of collagen scaffolds is known to modulate the lineage commitment and differentiation fate of MSCs. The molecular mechanism of stiffness sensing supported by in vitro studies is primarily governed by the aforementioned ECM-integrin-talinactin clutch system. When DPSCs bind to a substrate, the mechanical resistance of the collagen fibers exerts tension on focal adhesions. Substrate stiffness exceeding specific physiological thresholds (e.g., >5 kPa) triggers a force-dependent conformational change in the cytoskeletal protein talin, causing it to unfold and expose binding sites for vinculin. This physical maturation of focal adhesions is believed to lead to the rapid phosphorylation and downstream activation of Focal Adhesion Kinase (p-FAK) and Extracellular Signal-Regulated Kinase (p-ERK 1/2) [86]. Crucially, this cytoskeletal tension modulates the Hippo signaling cascade, leading to the dephosphorylation and subsequent nuclear translocation of the mechanosensitive transcription factors YAP (Yes-associated protein) and TAZ (transcriptional coactivator with PDZ-binding motif). When localized within the nucleus, YAP and TAZ interact bidirectionally with the canonical Wnt/β-catenin and BMP/Smad signaling pathways to orchestrate the targeted upregulation of osteogenic and odontogenic genes, including RUNX2, DSPP, and DMP-1. Conversely, exposure to highly rigid substrates (e.g., 28 kPa) forces robust YAP/TAZ nuclear accumulation, heavily biasing the DPSCs toward an odontoblast-like fate characterized by marked cellular elongation, matrix metalloproteinase (MMP) secretion, and the initiation of hard-tissue biomineralization [86]. One research team engineered two distinct collagen hydrogels with contrasting stiffness profiles—a soft formulation (Col^3^, 735 Pa) and a rigid one (Col^10^, 8142 Pa). The softer hydrogel preferentially directed DPSCs toward an endothelial lineage, as evidenced by upregulated expression of von Willebrand factor (vWF) and CD31. In contrast, the stiffer matrix enhanced odontogenic differentiation, marked by elevated levels of DSPP and RUNX2 [87]. These values strategically approximate the mechanical properties of native pulp (500–1000 Pa) and predentin (5000–10,000 Pa), respectively, supporting the concept that biomimetic physicochemical cues can guide stem cell fate toward specific tissue regeneration pathways [87].

Beyond pure elastic stiffness, native dental pulp tissue exhibits complex viscoelastic properties—it is not purely elastic but possesses a time-dependent mechanical profile characterized by stress relaxation and fluid-like viscous dissipation [88]. The viscosity of collagen scaffolds represents a critical design parameter for clinical translation in pulp regenerative therapy. When stem cells exert traction forces on a viscoelastic collagen matrix, the polymer network can physically rearrange, allowing the material to yield and dissipate stress over time. Studies manipulating the viscoelasticity of type I collagen hydrogels—independent of their initial stiffness—demonstrate that the rate of stress relaxation governs fundamental cellular processes. It has been observed that fast-relaxing soft gels are generally associated with restricted cell spreading and the maintenance of stemness, whereas slow-relaxing materials are often accompanied by more pronounced cell spreading, migration, and differentiation. This suggests that the viscoelastic timescale may serve as an important physical cue regulating the fate of dental pulp stem cells, although the underlying molecular mechanisms remain to be elucidated. A comparative in vitro study by V. Rosa et al. revealed significant temporal differences in odontogenic differentiation when stem cells from human exfoliated deciduous teeth (SHED) were cultured in Puramatrix™ versus recombinant human type I collagen (rhCollagen): the Puramatrix™ group exhibited odontoblast markers within 7 days, while the rhCollagen group required 14 days, which was attributed to rhCollagen’s higher viscosity, potentially hindering SHED migration and delaying the diffusion of dentin-derived morphogenetic signals [89]. These findings directly inform scaffold design strategies: optimizing viscosity parameters can enhance cell motility and accelerate bioactive signal propagation.

Collagens from different sources exert varying effects on pulp regeneration, depending on their structure, purity, and bioactivity. Currently, collagens applied in dental pulp regeneration are primarily derived from bovine or porcine skin and tendon tissues. These naturally sourced collagens retain intact spatial structures and biological information, providing a highly biomimetic microenvironment for cells [90]. The advantages of animal-derived collagens lie in their mature extraction processes and relatively low cost. However, significant differences exist in antigenicity and biocompatibility among collagens from different species. Bovine-derived collagens (such as commercial products like CollaPlug^®^ and CollaTape^®^) are the most widely used in dental pulp revascularization procedures. Meanwhile, porcine-derived collagens have garnered attention due to their higher structural similarity to human collagen. At the molecular level, the differences among collagens from various sources are primarily reflected in their capacity as cellular information carriers. Studies have demonstrated that mammalian collagens exhibit significantly stronger binding affinity to integrin α2β1 compared to fish collagens, with hydroxyproline (Hyp) content showing a positive correlation with binding capacity [91]. The GFOGER sequence (Gly-Phe-Hyp-Gly-Glu-Arg) in type I collagen serves as the key motif for specific binding to integrin α2β1 [92]. This binding triggers downstream osteogenesis-related signaling pathways (such as focal adhesion kinase [FAK], mitogen-activated protein kinase [MAPK], and runt-related transcription factor 2 [RUNX2]), thereby accelerating the osteogenic process. This specific interaction explains the notable differences in the efficacy of dental pulp regeneration observed with collagens from diverse sources.

**Figure 2:**
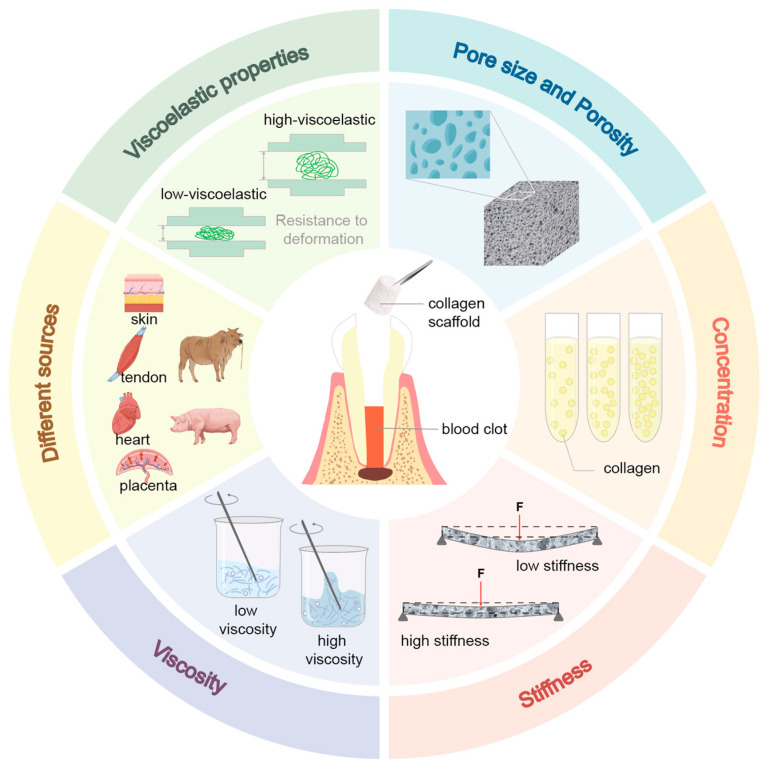
Schematic diagram illustrating the physicochemical properties of collagen scaffolds influencing the regeneration of the pulp-dentin complex. The blue section indicates the pore size and porosity of the collagen scaffold. Larger pore sizes or higher porosity facilitate the transport of nutrients and signaling molecules but tend to reduce mechanical strength. The orange section denotes the concentration of the collagen scaffold. Excessively low concentration leads to pronounced scaffold contraction, whereas overly high concentration restricts cell migration and proliferation. The red section represents the stiffness of the collagen scaffold. Scaffolds with low stiffness preferentially guide the differentiation of dental pulp stem cells toward an endothelial lineage, while those with high stiffness enhance odontoblastic differentiation. The purple section refers to the viscosity of the scaffold. Excessive viscosity impedes cell migration and the propagation of bioactive signals. The yellow section highlights the diverse sources of collagen scaffolds, which can be derived from animal skin, tendon, heart, and placenta. Collagens from different species exhibit significant differences in antigenicity and biocompatibility. The green section describes the viscoelastic properties of collagen scaffolds. Dynamic and compressive characteristics that closely resemble those of native dental pulp are more favorable for pulp regeneration.

Moreover, the viscoelastic properties of collagen scaffolds are closely related to pulp regeneration (Figure 2). Through experimental measurements and analyses, Cevat Erisken et al. demonstrated that the dynamic properties of collagen gels, including storage modulus, loss modulus, and tan δ, approximated those of natural pulp tissue, although their compressive properties fell significantly short [10]. Based on these findings, the study proposed that collagen scaffolds and other regenerative materials should more accurately replicate both the dynamic and compressive properties of natural pulp by modulating gelation agents and their concentrations, in order to enhance regenerative efficacy.

Despite its well-documented biomimetic advantages, the application of collagen in dental pulp regeneration is limited by inherent biological and physical constraints, regarding degradation kinetics and mechanical vulnerability. Unmodified type I collagen undergoes unpredictable enzymatic degradation within the proteolytic environment of the root canal, primarily via cleavage by endogenous MMPs and bacterial collagenases. This accelerated bioresorption frequently results in premature scaffold collapse before neovascularization and extracellular matrix deposition by stem cells reach functional sufficiency, thereby halting regeneration and leaving an empty canal space [93]. In its native, uncrosslinked state, collagen also lacks the mechanical integrity required to withstand physiological forces; upon hydration, it exhibits poor dimensional stability and structural weakness, rendering it prone to collapse. Such collapse occludes the interconnected porous architecture, thereby obstructing angiogenesis and nutrient perfusion [94]. Furthermore, standard sterilization methods further compromise its bioactivity by denaturing the triple-helix structure and disrupting critical motifs such as RGD sequences [94]. These limitations, together with residual immunogenic risks and regulatory concerns, hinder the translational potential of pure collagen scaffolds [94].

In conclusion, owing to their natural cell adhesion motifs (such as RGD sequences), favorable porous networks, and dynamic viscoelasticity, collagen scaffolds efficiently mediate stem cell adhesion and guide their directed differentiation, demonstrating remarkable bioactivity and biomimetic advantages. Compared with other scaffolds commonly used in regenerative endodontic procedures (REPs), synthetic polymers (such as PLGA and PCL) can provide highly tunable, robust mechanical support and predictable degradation cycles through engineering approaches, thus exhibiting superior physical stability [94]. But it lacks bioactive components, resulting in low cell affinity. PRF contains leukocytes and platelets embedded within a fibrin network, which not only enables the release of antimicrobial peptides and anti-inflammatory factors that help control infection and modulate immune responses, but also allows sustained and stable release of high concentrations of key growth factors such as TGF-β1, PDGF-BB, and VEGF, directly promoting stem cell homing, differentiation, and angiogenesis [95]. However, its limitations are evident, including substantial variability in quality depending on donor characteristics and processing methods, and excessively rapid degradation rates [96]. Additionally, decellularized ECM scaffolds can preserve donor tissue-specific complex three-dimensional topological structures and comprehensive natural biochemical signals alongside other bioactive components, offering significant advantages in both spatial structural complexity and biological information richness [97]. However, its mechanical properties are unstable and difficult to control, preventing large-scale production and application. Taken together with the aforementioned studies, the physicochemical properties of the collagen scaffold are essential in modulating the formation of the pulp-dentin complex. While initial studies suggested that an optimal collagen scaffold for pulp regeneration might possess a pore size of approximately 60–90 μm and a porosity of around 95%, contemporary evidence demonstrates that such rigid design-level specifications fail to account for the complex, stage-specific requirements of tissue neogenesis. Current consensus acknowledges that these parameters interact dynamically, necessitating hierarchical or gradient architectures rather than a single uniform pore size or porosity threshold [78]. Furthermore, studies suggest that mimicking the hardness, viscosity, and viscoelasticity of native dental pulp tissue enhances pulp regeneration, underscoring the importance of “biomimicry of materials” as a critical area for exploration. Additionally, the degradation rate of the scaffold should synchronize with the rate of new pulp tissue formation. However, current research has limitations. On the one hand, the interactions between different physicochemical properties remain unclear. The efficacy of collagen scaffolds in promoting pulp regeneration stems from the synergistic effects of multiple physicochemical properties; thus, a systematic consideration of multiple physicochemical indicators is necessary when evaluating the potential of collagen scaffolds. On the other hand, the optimal concentration range of collagen scaffolds and data on pulp tissue regeneration rates require further investigation. Such research would guide the construction of collagen scaffolds with appropriately synchronized degradation rates, ultimately improving clinical efficacy. Considering the respective advantages of alternative scaffold materials, to enhance regeneration outcomes, the physicochemical properties of collagen scaffolds can be enhanced through the following approaches to improve pulp regeneration outcomes: (1) optimizing extraction and fabrication processes such as utilizing electrospinning or 3D bioprinting [93]; (2) modifying collagen scaffolds via surface treatment or chemical functionalization; and (3) incorporating other materials into pure collagen to form composite scaffolds to overcome inherent mechanical weaknesses [94]. Ultimately, development in tissue engineering for pulp regeneration has garnered increasing attention. By utilizing collagen scaffolds as carrier vehicles, this approach holds significant promise for transcending the inherent limitations of the scaffolds themselves.

## 6. Advancing Collagen Scaffolds Through Tissue Engineering for Precision Regeneration

Regeneration of the pulp-dentin complex involves a cascade of biological events, including bioactive molecule activity, stem cell recruitment, angiogenesis, and neurogenesis, that remain difficult to achieve using current REPs [98]. Dental pulp tissue engineering, based on the “cell–scaffold–biomolecule” triad, provides a theoretical and practical framework for “toward the goal of controllable ‘true regeneration’” [99]. However, this goal requires further research to optimize collagen scaffold design and integrate tissue engineering strategies.

Collagen scaffold functions both as reservoirs and sustained-release carriers of bioactive molecules, and as matrices that encapsulate stem cells to enhance pulp regeneration. Studies indicate that incorporating stem cells or biomolecules into collagen scaffolds represents an approach to achieve genuine regeneration of the pulp-dentin complex [87,100,101]. A comparative experiment demonstrated that the combined use of hDPSCs, type I collagen scaffolds, and DMP1 significantly promoted differentiation into odontoblast-like cells, collagen matrix deposition, and angiogenesis, compared with collagen-based scaffolds alone [100]. Similarly, another in vivo mouse experiment showed that collagen scaffolds loaded with VEGF and bone morphogenetic protein 2 (BMP2) stimulated DPSCs to differentiate into endothelial cells and odontoblasts, and successfully generated blood-perfused vascular networks and mineralized dentin tissue [87]. As reviewed by Farjaminejad et al., collagen scaffolds loaded with VEGF and BMP-2 can synergistically induce DPSC differentiation into endothelial cells and odontoblasts, successfully regenerating tissues containing blood-perfused vascular networks and mineralized dentin in vivo [5].

In addition to the biomolecules mentioned above, other critical signaling molecules, including Wnt, transforming growth factor-β (TGF-β), Ectodysplasin A (EDA), Sonic Hedgehog (SHH), and members of the MAPK family, also play crucial roles in dental pulp regeneration [102] and were summarized in Table 3. It is worth noting that microRNAs exert multiple regulatory roles in dental pulp-dentin regeneration. Future approaches combining collagen scaffolds with stem cells and specific miRNAs, such as miR-378 inhibitors or miR-26b/miR-126-3p, may achieve synergistic effects, enabling advanced functional regeneration of the pulp-dentin complex [103]. However, traditional direct loading often suffers from burst release and rapid inactivation of growth factors. To address this, researchers have developed nanomaterial-mediated sustained-release systems. For instance, the use of laponite to adsorb G-CSF before combining it with collagen achieves a sustained release profile, significantly improving the recruitment efficiency and odontogenic differentiation of SCAPs [101]. This aligns with the concept of “smart scaffolds” that utilize nanoparticles, gene-functionalized molecules, or responsive hydrogels to achieve spatiotemporal control over growth factor release, thereby precisely regulating angiogenesis, stem cell differentiation, and the immune microenvironment [104,105,106].

To further enhance the regenerative capacity of collagen scaffolds beyond simply combining them with stem cells and growth factors, advanced functionalization strategies are being actively explored, such as the incorporation of bioactive peptides and mineralized components. Bioactive peptide modification offers a more stable and cost-effective alternative to whole growth factors. By mimicking the functional domains of native proteins, these peptides exhibit lower immunogenicity and higher stability. For example, synthetic peptides derived from BMP-2 have been loaded into mineralized collagen composites, where they not only achieve sustained release but also significantly upregulate the expression of odontogenesis-related genes, inducing reparative effects comparable to recombinant human BMP-2 in animal models [107]. In the dental field, RGD peptide-containing recombinant collagens have been shown to enhance the adhesion, migration, and odontogenic differentiation of mesenchymal stem cells [108]. This strategy holds promise for developing more precise and safer cell-free therapies by screening or designing homing peptides or odontogenic induction peptides that specifically target SCAPs or DPSCs. Mineralized component incorporation aims to replicate the biomineralized microenvironment of native dental tissues. Compositing mineral components such as HA, β-tricalcium phosphate (β-TCP), or bioactive glass with collagen not only improves the mechanical properties of the scaffold but also provides biophysical cues conducive to odontoblast differentiation. As reviewed by Thirumalaivasan [93], mineralized collagen composite scaffolds regulate stem cell fate through surface topography and ion release—magnesium-doped mineralized recombinant collagen scaffolds significantly promoted the migration and osteogenic differentiation of mesenchymal stem cells. More notably, the strategy of inducing intrafibrillar mineralization of collagen using genetically engineered peptides can mimic the nanostructure of natural dentin. Ma et al. utilized enamel hydroxyapatite binding peptide (EHABP) to mediate the infiltration of amorphous calcium phosphate into collagen fibrils, achieving highly biomimetic intrafibrillar mineralization [109]. This technology holds significant promise for the future construction of dentin regeneration scaffolds with excellent structure and function.

Stem cells constitute one of the three essential elements in tissue engineering. Cells utilized for pulp regeneration can originate from various odontogenic and non-odontogenic sources. Non-odontogenic sources include embryonic stem cells, neural crest stem cells, BMMSCs, adipose-derived MSCs (AD-MSCs), and umbilical cord MSCs (UC-MSCs) [110]. Odontogenic stem cells, including DPSCs, SHEDs, and stem cells from apical papilla (SCAPs), exhibit multilineage differentiation abilities, making them key candidates for pulp-dentin complex regeneration. DPSCs display typical characteristics of MSCs [111] and demonstrate lower immunogenicity and higher proliferation rates compared with other MSCs, highlighting their potential for regenerative applications [112,113]. Liu et al. recently demonstrated that injection of allogeneic DPSCs significantly promotes periodontal regeneration in a clinical trial, indicating the maturation and clinical relevance of DPSC-based therapies [114]. Within a collagen scaffold, DPSCs are particularly effective at maintaining pulp homeostasis and promoting vascularization through paracrine signaling, as demonstrated in 3D culture systems where they support endothelial cell migration and tubule formation [115]. SCAPs, derived from developing roots, possess robust proliferation and differentiation capacities, facilitating apex closure and root development. Compared with DPSCs, SCAPs exhibit higher telomerase activity and greater dentinogenic potential, and are more likely to express odontogenic markers like DSPP and DMP-1 rather than osteogenic markers when appropriately induced, making them ideal for true dentin regeneration [116]. This inherent bias towards an odontogenic lineage, combined with their natural role in root development, makes SCAPs particularly valuable for regeneration in immature teeth. The extracellular matrix-mimicking environment of a collagen scaffold provides an ideal niche for SCAPs, and strategies utilizing chemotactic factors like G-CSF have been shown to specifically recruit host SCAPs, leading to successful regeneration of vascularized pulp-like tissues in animal models [101]. Although SCAPs remain functional in normal or reversibly inflamed pulp tissues, irreversible pulpitis decreases cell viability, and necrosis results in tissue disintegration [69]. Furthermore, the study demonstrates that SHEDs are stem cells characterized by strong proliferative ability, multilineage differentiation (particularly advantageous for synergistic neural and vascular regeneration), and notable immunomodulatory properties, making them suitable for functional pulp-dentin regeneration [75]. Despite their critical role in REPs, ethical concerns and limitations regarding differentiation potential remain issues for stem cells [117]. Recently, cell-free therapies have emerged, utilizing exosomes derived from odontogenic stem cells. These exosomes carry bioactive components, such as miRNAs, proteins, and lipids, inherited from parent cells, effectively mimicking stem-cell paracrine effects [118]. Animal experiments have demonstrated that exosome treatments enhance reparative dentin bridge formation and support regeneration of vascularized pulp-dentin tissues, including structures resembling dentinal tubules and odontoblast-like cells [103,119]. This approach promotes dental pulp regeneration while addressing ethical, safety, and preservation challenges associated with traditional cell-based transplantation.

Although research involving collagen scaffolds combined with stem cells and growth factors has demonstrated regeneration of vascularized and innervated pulp-like tissues [120,121], there remains a lack of systematic approaches for reconstructing neural networks, reliable in vivo/in vitro experimental models, and methods for functional nerve assessment. Re-innervation plays a crucial role in pulp healing, whereas denervation impedes dentin formation [122,123]. These findings highlight the critical importance of neural regeneration for restoring the neurovascular function. However, limited neural regeneration in regenerative pulp models, unlike in pulp injury models, may result from the absence of a functional stem cell niche capable of secreting the necessary neurotrophic factors. In rat experiments, collagen scaffolds combined with rBMSCs, VEGF, and fibroblast growth factor 2 (FGF2) increased the expression of neuron-specific markers (S-100, PGP9.5). They facilitated the formation of neuron-like cells and nerve terminals within regenerated tissue [120,121]. MSCs, such as DPSCs, can undergo neural differentiation, adopting Schwann cell-like phenotypes and expressing elevated levels of neural markers, including paired box gene 6 (PAX6), Nestin, and β-III-tubulin, which support neurogenesis [124,125]. This strategy represents an emerging direction in peripheral nerve injury (PNI) repair [126]. However, the mechanisms underlying neurogenic processes after REPs remain poorly characterized, potentially explaining the low success rates of pulp vitality restoration. Thus, re-establishing neural structures is an essential goal in regenerative endodontics.

Furthermore, their broad clinical translation of collagen scaffolds in REPs is restricted by regulatory, clinical, and practical barriers. For regulatory compliance, these dental biomaterials must adhere to stringent international medical device standards, including ISO 10993 for comprehensive biological safety evaluation, FDA 510(k) clearance pathways in the United States, and CE marking requirements for clinical commercialization in the European Union, benchmarks that guide the development and validation of novel scaffold formulations. Clinically, successful implementation demands standardized protocols for scaffold handling, precise root canal placement, and integration with effective endodontic disinfection strategies to prevent treatment failure secondary to persistent infection. Key translational bottlenecks include insufficient long-term multicenter clinical evidence, batch-to-batch consistency variability in custom scaffold fabrication, and elevated production costs limiting routine clinical use. These barriers can be mitigated through industrialized manufacturing standardization, large-scale prospective clinical trials, and multidisciplinary consensus guidelines developed by endodontic and biomaterials research bodies. Closing these critical gaps is imperative to bridge preclinical laboratory innovation and bedside clinical application, fully unlocking the capacity of collagen scaffolds to revolutionize regenerative endodontic care.

In summary, regeneration of the pulp-dentin complex is a sophisticated spatiotemporal process. Most commercial collagen scaffolds used in REPs are derived from non-pulp tissues. These materials lack adequate biomimetic design, provide insufficient odontogenic induction, and offer limited support for neurovascular regeneration. Consequently, current collagen-based REPs typically result in tissue repair rather than true functional regeneration. Incorporating pulp-specific bioactive factors (e.g., BMPs, TGF-β, FGFs) and stem cells into collagen scaffolds via tissue engineering strategies may therefore facilitate genuine pulp-dentin regeneration. The distinct roles of DPSCs and SCAPs further highlight the demand for cell-type-specific scaffold designs. Future developments in advanced collagen scaffolds should focus on integrating multiple functionalization strategies—such as the synergistic combination of growth factors, bioactive peptides, and mineralized components—to enable spatiotemporal control of the release of crucial biomolecules and better replicate the native ECM [104,127]. Furthermore, exploiting the paracrine capabilities of stem cells via cell-free therapies, such as exosomes or engineered extracellular vesicles containing pro-regenerative miRNAs and growth factors, may resolve challenges associated with cell sourcing, storage, and immunogenicity. However, foundational research on pulp regeneration remains limited, particularly regarding neuro-immune-vascular interactions within the pulp microenvironment. This gap in knowledge restricts the innovative design of novel collagen scaffolds for REPs. Achieving fully functional, innervated, and vascularized pulp-dentin regeneration requires interdisciplinary collaboration among material scientists, biologists, and clinicians.

**Table 3:** Key signaling molecules in the formation of the dentin-pulp complex.

Signaling Factor	Subtype	Regenerative Function
BMPs	BMP2	Promotes odontoblastic differentiation of DPSCs [128] and facilitates dentin formation; promotes pulp angiogenesis via VEGF-A production in differentiated odontoblasts [129].
	BMP4	Enhances odontoblast differentiation capacity [130].
	BMP7	Promotes the transformation of DPSCs to a mineralized phenotype [131].
	BMP9	Promotes the differentiation and secretory function of dental pulp stem cells; it enhances cell proliferation and intercellular connections in HERS, thereby facilitating the formation of root dentin and the closure of the apical foramen [132].
TGF-β	TGF-β1	Guides cell migration, proliferation, and differentiation; stimulates odontoblast-like cells to secrete DSPP [133,134].
	TGF-β2	Upregulates odontogenic markers (DSPP, DMP1) and suppresses osteogenic marker bone sialoprotein in SCAPs [135].
	TGF-β3	Promotes odontoblastic differentiation [136].
FGFs	FGF-2	Regulates all stages of tooth development [137], repair, and regeneration [138], including migration, proliferation, stemness maintenance of mesenchymal stem cells, dentin formation, angiogenesis, and neurogenesis [139].
VEGF		Induces endothelial differentiation of stem cells and regulates tooth development and dentin formation [140].
IGF	IGF-1	Promotes proliferation and differentiation of DPSCs and SCAPs [141], and induces their transformation to a mineralized phenotype [142].
miRNA		Regulates expression of key odontogenic differentiation markers; promotes neurogenesis; enhances endothelial differentiation and supports vascular regeneration [103].
EGF		Enhances neurogenic differentiation of DPSCs [143] and SCAPs [144].
NGF		Promotes neurite outgrowth and neural cell survival; critical for the maintenance of sympathetic and sensory neurons [145,146].

Abbreviations: BMP: bone morphogenetic protein; DPSCs: dental pulp stem cells; HERS: Hertwig’s epithelial root sheath; TGF-β: transforming growth factor beta; DSPP: dentin sialophosphoprotein; DMP1: dentin matrix protein 1; SCAPs: stem cells from the apical papilla; FGF: fibroblast growth factor; VEGF: vascular endothelial growth factor; IGF-1: insulin-like growth factor-1; miRNA: microRNA; EGF: epidermal growth factor; NGF: nerve growth factor.

## 7. Conclusions

Collagen scaffolds represent a potential biomaterial for advancing the field of regenerative endodontics. Their inherent biocompatibility, biodegradability, and ability to mimic the native extracellular matrix provide a conducive microenvironment for the recruitment, proliferation, and differentiation of stem cells, which is essential for the regeneration of the pulp-dentin complex. Evidence from clinical studies demonstrates that collagen scaffolds can support high survival rates and promote desirable outcomes, such as pulp vitality recovery, dentin wall thickening, and apical closure in necrotic immature permanent teeth. However, they are not particularly adept at facilitating canal length. The formation of heterogeneous mineralized tissues—primarily cementum-like and bone-like tissues rather than genuine reparative dentin—highlights that current REPs using collagen scaffolds often result in repair rather than true physiological regeneration. Most collagen scaffolds currently used in clinical or experimental settings are repurposed from periodontal or implant dentistry and are not designed for pulp regeneration. Thus, translating collagen-based strategies into predictable clinical protocols poses several challenges. Here, we propose three targeted design principles for next-generation collagen scaffolds dedicated to genuine dentin regeneration: developing composite materials to enhance mechanical properties and control degradation kinetics, and meticulously optimizing key physicochemical parameters (e.g., pore size, stiffness, viscosity) to create a truly biomimetic microenvironment; integrating dental tissue engineering approaches, including seeding stem cells or incorporating bioactive molecules (e.g., miRNAs) to further guide authentic pulp tissue regeneration; advancing cell-free strategies, such as employing exosomes or engineered extracellular vesicles loaded with pro-regenerative miRNAs and growth factors, which could harness the paracrine potential of stem cells while overcoming challenges related to cell sourcing, storage, and immunogenicity. These principles will guide the rational development of advanced collagen-based biomaterials, bridging preclinical research and clinical translation to enable predictable, true pulp-dentin regeneration in immature necrotic teeth. By addressing these challenges through interdisciplinary collaboration, collagen scaffolds have shown potential to advance regenerative endodontics, as they support high survival rates, dentin wall thickening, and apical closure in necrotic, immature permanent teeth. However, current evidence indicates that they often induce repair (formation of cementum-like or bone-like tissue) rather than true pulp-dentin regeneration. To realize their potential for achieving fully functional, vascularized, and innervated pulp-dentin complex regeneration, future work must focus on optimizing collagen scaffold design to enhance odontogenic induction, neurovascular regeneration, and mechanical stability, and on conducting rigorous long-term clinical studies to validate these improvements.

## Data Availability

No new data were created or analyzed in this study.
